# Influence of gravity on water management and mass transport losses in polymer electrolyte membrane fuel cells

**DOI:** 10.1038/s41598-025-09067-y

**Published:** 2025-11-11

**Authors:** Eric A. Chadwick, Beste Derebaşı, Volker P. Schulz, Aimy Bazylak

**Affiliations:** 1 Bazylak Group, Department of Mechanical & Industrial Engineering, Faculty of Applied Science and Engineering, University of Toronto, 5 King’s College Road, Toronto, ON M5S 3G8 Canada; 2 Electrochemical Cluster, Department of Mechanical Engineering, Baden-Württemberg Cooperative State University, Mannheim, Baden-Württemberg Germany

**Keywords:** Gravity, Polymer electrolyte membrane fuel cells, Radiography, Distribution of relaxation times, Mass transport resistance, Liquid water management, Fuel cells, Mechanical engineering

## Abstract

Traditionally, gravity has been assumed to have a negligible impact on the multiphase transport behaviour in polymer electrolyte membrane (PEM) fuel cells; however, in this study, we reveal how the impacts of gravity and flow field orientation should not be ignored. Gravity-assisted reactant flow provides up to a 21.2 % higher peak power density compared to gravity-opposed reactant flow, owing to enhanced water removal (which we observed via *operando* synchrotron radiography). We are the first to combine a distribution of relaxation times (DRT) analysis with *operando* imaging, and for the first time, we separately correlate the presence of liquid water in the channels and GDLs to distinct mass transport loss contributions via this approach. Liquid water accumulation in the cathode GDL is most typically the focus of water management in the PEM fuel cell; however, in this work, we observed significant water accumulation in the anode GDL and channels at gravity-opposed orientations. Specifically, we observed large droplets and slugs in the anode channels which led to significantly higher anode GDL water saturation (≥ 0.23) compared to gravity-assisted angles (≤ 0.11). The force of gravity overpowers the weaker inertial force of reactant hydrogen flow, thereby hindering the removal of water droplets in the anode flow fields, which results in poor water management, reactant starvation and ultimately cell failure.

**Supplementary Information:**

The online version contains supplementary material available at 10.1038/s41598-025-09067-y.

## Introduction

The existential threat of climate change has motivated many nations to urgently reduce carbon emissions in an effort to mitigate global temperature rises caused by greenhouse gas emissions^1,2^. Given that transportation accounts for one fifth of global carbon emissions worldwide^3,4^, replacing carbon-emitting vehicles with battery-electric or fuel cell-electric vehicles is imperative. Due to their high energy density relative to batteries^5^, polymer electrolyte membrane (PEM) fuel cell-electric vehicles are well-suited for use in heavy-duty vehicles such as long-haul trucks^6,7^, naval ships^8^, and commercial aircrafts^9^. However, fuel cells still face significant challenges in widespread commercialization in these applications. Based on United States Department of Energy (DOE) targets, to be competitive with other technologies in heavy-duty applications, fuel cells must be improved to increase cell lifetimes by 20 %, reduce cell costs by 25 %, and improve efficiency by 4 % by the year 2050^7^.

PEM fuel cells provide power by converting hydrogen and oxygen gas into liquid water. Optimized liquid water management in PEM fuel cells is key to reaching commercial targets. Adverse liquid water accumulation in the channels and gas diffusion layer (GDL) of a PEM fuel cell can hinder reactant delivery to the catalyst layer (CL) reaction sites, thereby limiting the power density and energy efficiency of the fuel cell, which increases energy costs^10^. Moreover, significant liquid water flooding causes reactant starvation, which promotes CL carbon corrosion, reduces catalyst surface area, and degrades CL lifetimes^11–14^. In recent years, *operando* imaging has been applied by a few groups worldwide, and they have provided important insights into how operating conditions^15–18^ and material design^19–22^ impact liquid water accumulation and electrochemical performance. However, the typically small active areas used for these specialized *operando* experiments have not been particularly complementary to the study of how external body forces impact operation. To commercialize PEM fuel cells for transportation, it is critical to understand how external body forces, such as gravity, vehicle acceleration, and impact-induced vibrations affect performance.

The impact of gravity is often ignored in PEM fuel cell modelling and experiments due to the presumed low Bond number in the membrane electrode assembly (MEA) and channels. However, a recent study by Ayaz et al. experimentally demonstrated that even for Bond numbers less than one, the orientation of a porous material can significantly impact drainage patterns (e.g. displacement of water by air)^23^. Furthermore, in our previous work, we used numerical modelling to show how gravity can significantly impact saturation levels in drainage scenarios in stochastically generated porous media, even for Bond numbers as low as 10^− 4^ (corresponding to a mean pore size of 30 μm at 25 °C and 1 atm)^24^. In the literature, gravity has been shown to significantly deteriorate performance in single cell operation^25^ and especially in stack operation^26^ due to increased water accumulation in the flow field channels when reactant flow is gravity-opposed. To verify the effects of cell orientation on liquid water accumulation, optical imaging techniques have been employed on operating fuel cells with transparent flow field plates^25,27–29^. For example, Ashrafi et al. showed via optical and numerical methods that more water accumulates in cathode channels when flowing reactants against gravity^28^. Liu et al. also demonstrated a correlation between liquid water in the anode channels and local current density at various orientations^29^. These past works provide valuable insight into the effect of gravity on channel liquid water accumulation; however, these studies have been focused on identifying liquid water in the flow field channels. The impact of gravity on water transport behaviour across the entire fuel cell has not yet been presented in the literature. Furthermore, the study of liquid water in the PEM fuel cell has been primarily focused on the cathode, leaving a scarcity of information regarding water accumulation in the anode despite the prevalence of anode liquid water in fuel cells with large active areas^30–32^. Spendelow et al. evaluated the impact of cell orientation on liquid water in the entire cell assembly^33^ using neutron radiography, and they found that placing the anode flow field above the cathode led to decreases in liquid water accumulation in the anode and cathode; however, to-date, vertical orientations or other angles have not been discussed in the literature.

While the previously mentioned studies^25,27–29,34^ have visualized the effects of gravity on liquid water accumulation, few have coupled these effects to electrochemical losses while imaging. Correlating liquid water accumulation obtained using imaging techniques to polarization losses is often accomplished with electrochemical impedance spectroscopy (EIS) in combination with equivalent circuit modelling^35–38^. However, multiple equivalent circuit models can be valid for the same fuel cell system, and the multiplicity of solutions leads to some ambiguity regarding the interpretation of EIS spectra^39^. For example, the use of Warburg components has been previously shown to erroneously attribute the bulk of electrochemical losses to diffusion limitations in systems where charge-transfer losses are actually dominant^40^.

Recently, distribution of relaxation times (DRT) analysis has emerged as a more advanced form of equivalent circuit modelling. In electrochemical cells where resistive-capacitive features dominate the total cell impedance (such as in PEM fuel cells), DRT analysis can be used to distinguish loss mechanisms as peaks in cell resistance as a function of time constants or frequency^41–49^. Unlike equivalent circuit modelling, DRT remains valid in the same system with altered parameters such as inlet RH conditions and cell temperature, making DRT a more robust technique. Kulikovsky has extensively explored DRT analysis in PEM fuel cells^41–46^, showing numerically that the activation losses of the anode and cathode half reactions can be distinguished from mass transport losses due to significant differences in time constants of each mechanism. In particular, Kulikovsky employed DRT to demonstrate that mass transport resistance peaks can manifest at different frequencies corresponding separately to the GDL, CL, or flow field channels^45,46^. Additionally, Bevilacqua et al. and Weiss et al. demonstrated that calibration experiments can be used to attribute specific DRT resistance peaks to reactant mass transport^47,48^. A comprehensive study of DRT in low-temperature PEM fuel cells by Heinzmann et al. demonstrated that mass transport loss resistance in the anode and cathode occur at similar frequencies and found that lower hydrogen partial pressure increased the relative contributions of anode mass transport losses^49^. These studies have advanced the understanding of the sensitivity of certain losses to changes in operating conditions; however, to date, no study has combined DRT analysis with *operando* imaging to directly correlate liquid water accumulation to mass transport losses in specific cell components. Moreover, there is a knowledge gap in understanding the effects of gravity on specific mechanisms of losses in PEM fuel cells that can be probed via concurrent *operando* imaging and DRT analysis.

In this study, to determine the effect of gravity on liquid water accumulation in the reactant flow channels and MEA of both the cathode and anode of a PEM fuel cell, we employed *operando* synchrotron X-ray radiography at 6 distinct angles. We simultaneously employed electrochemical testing and EIS followed by DRT analysis at each angle, to draw connections between specific mechanisms of overpotential loss and liquid water accumulation. This study also marks the first combination of *operando* imaging and DRT analysis, enabling the direct correlation of water distributions in the fuel cell to specific loss mechanisms revealed in the DRT response.

## Methods and materials

We conducted *operando* synchrotron radiography on a PEM fuel cell positioned at various angles relative to the imaging stage using a custom-built positioning fixture, and we examined the impact of gravity on the electrochemical performance and liquid water accumulation of the PEM fuel cell. The details of the electrochemical testing and electrochemical impedance spectroscopy (EIS) are described in the “Electrochemical testing” section. Next, the positioning fixture and imaging set-up are detailed in the “Quantification of liquid water” section. Finally, to accurately quantify the effect of liquid water on specific mass transport phenomena at each angle, we employed distribution of relaxation times (DRT) analysis on the EIS spectra in the “Dimensionless numbers” section.

### Electrochemical testing

#### Cell hardware and fuel cell materials

A long flow path length was desired to visualize the effects of gravity on water transport in the PEM fuel cell. To achieve this, parallel flow fields with four 1 mm-wide × 1 mm-deep × 40 mm-long channels were machined into graphite plates, and each channel was separated by a 1 mm-wide land. The active area of the fuel cell was 3.2 cm^2^ (40 mm-long × 8 mm-wide). For the gas diffusion layer (GDL), a 370 μm-thick Toray carbon paper (TGP-H-120) with a 60 μm-thick MPL (total thickness of 430 μm) (Fuel Cell Store, College Station, TX) was employed on both the anode and cathode. We used this thick GDL to intentionally establish a long liquid water path from the catalyst layer (CL) to the flow fields to increase the likelihood of observing the effects of gravity within the GDL. Sandwiched between the GDLs, the membrane electrode assembly (MEA) included a catalyst-coated membrane (CCM) consisting of a 20 μm-thick Nafion HP membrane symmetrically coated with 0.3 mgPt cm^−2^ (IonPower, New Castle, DE). Compression of the GDLs by approximately 20 % of the original GDL thickness is preferred for providing optimal contact without adversely affecting other resistances in the fuel cell^50^ PTFE) gasket at both the anode and cathode, compressing both GDLs by 18.6 % of their original thickness (430 ${\upmu}$m) (noting that we were intentionally targeting a compression of 20 %).

**Fig. 1:**
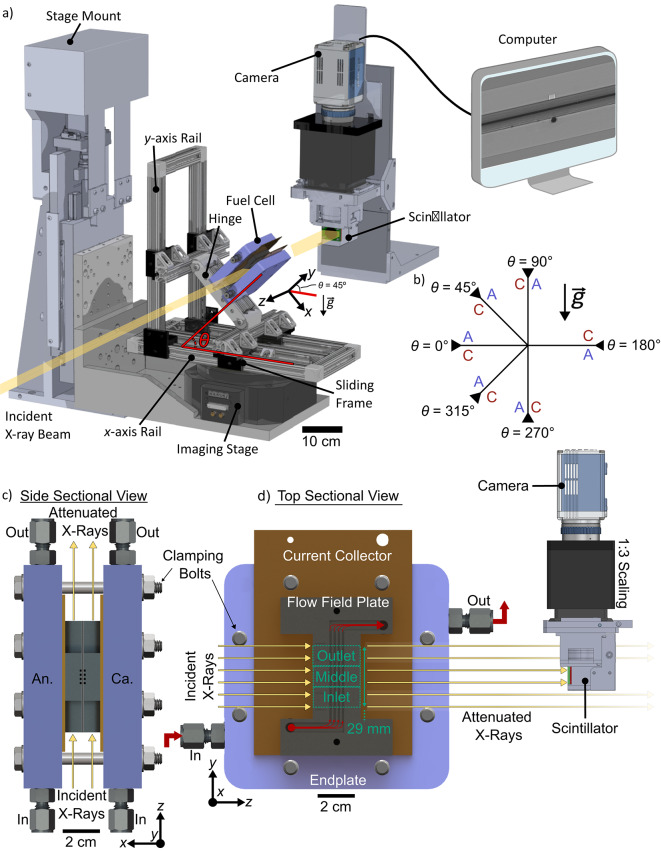
**a** Schematic of imaging set-up with the rotation fixture interfacing with the CLS stage and the operando fuel cell shown in the 45°/315° position (rotated about the z-axis—i.e. the beam). **b** Description of fuel cell angles in terms of hydrogen and air co-flow direction (indicated by arrow direction) and locations of the anode (A) and cathode (C) with respect to each other (i.e. one on top of the other). **c** Side sectional and **d** top sectional views of the operando fuel cell in the 270° position with labelled components and channels running perpendicular to the x-ray beam. In **d** we illustrate how the active area can only be observed within the 29 mm space between the attenuating clamping bolts. The region of interest (ROI) captured by the scintillator is highlighted with dashed lined in **d**. 3D models were created in SolidWorks 2022 (https://www.solidworks.com, Dassault Systèmes, Vélizy-Villacoublay, France).

Three fuel cell assemblies were each electrochemically tested at 6 distinct angles: 0°, 45°, 90°, 180°, 270°, and 315° (illustrated in Fig. 1a, b). Additional angles (e.g. 135° and 225°) were not tested due to limited beam time. For each assembly, only the anode and cathode GDLs and CCM were changed, such that the exact same end plates, current collectors and flow field plates were used for each assembly. Between assemblies, the thickness of PTFE gaskets was measured to ensure plastic deformation had not occurred before reusing them for the next assembly. For two of these assemblies, the experiments were conducted three times per angle for repeatability, and one of the assemblies was imaged at each angle without repetition due to time constraints during competitive beamtime for a total of 42 experiments (7 repetitions × 6 angles). To establish the angle of the fuel cell between experiments, a custom positioning fixture was designed (Fig. 1a). The fuel cell angle, *θ*, is formed by the *x*-axis rail and the fuel cell end plate, as shown by the red lines in Fig. 1a. The gravity fixture provides cell rotation between 0° and 90° while keeping the region of interest in the beam path without obstructions (Fig. 1a). To achieve angles 270° and 315°, the direction of the co-flow reactants was reversed (i.e. the inlet became the outlet and vice versa). To achieve the 180° angle, the gravity fixture was set to the 0° position, and the cell was turned upside down such that the cathode was above the anode. In Fig. 1b, the reactant co-flow direction is denoted by the arrows adjacent to the value of the angle, *θ*, and the location of the cathode and anode is denoted by C (in red) and A (in blue), respectively.

#### Experimental conditions

In practice, large-scale PEM fuel cells and stacks are known to exhibit high relative humidity near the outlet of cells^51^ due to reactant depletion along the path length of the flow fields—especially since low stoichiometric ratios are employed in practice (1–3 for cathode, 1–2 for anode^52,53^. Therefore, we supplied fully humidified (100 % relative humidity (RH)) hydrogen (99.99 % H_2_) and air to the anode and cathode, respectively. Inlet gases were supplied at a flow rate of 0.5 slpm in a co-flow arrangement controlled by a fuel cell test station (Scribner 850e, Scribner Associates Inc, Southern Pines, NC), resulting in an average reactant velocity of 2.08 m s^− 1^ in each channel, which is similar to what is applied in large-scale fuel cells^52,53^. This flow rate resulted in a minimum stoichiometry for hydrogen and air of 18.2 and 7.63, respectively. Cell temperature was held constant at 60 °C by electrically heated rods in the end plates of the cell, monitored by a T-type thermocouple in the cathode end plate and controlled by the fuel cell test station.

For each fuel cell assembly, the CCM was conditioned via voltage cycling between OCV, 0.6 V, and 0.3 V for 60 s at each voltage over a total period of 3 h (following the methods recommended by Klug et al. from Scribner Associates, Inc.^54^, using the same cell temperature and inlet conditions described in the previous paragraph (100 % RH, 0.5 slpm co-flow, cell temperature of 60 °C). Polarization curves were acquired during each experiment by first obtaining OCV at 0.00 A cm^− 2^ for 5 min, followed by 20-minute holds of 0.25, 0.50, 0.60, 0.70, and 0.80 A cm^− 2^ with ramps of 0.001 A s^− 1^ between each current density. The cell voltage was monitored during current ramping and during constant current testing, and if the voltage fell below 0.1 V or became unstable, the test was terminated (having reached cell failure conditions). The cell was purged of liquid water at 0.5 slpm co-flow using, humidified N_2_ gas (99.9 % purity) for 7 min, humidified fuels (H_2_ and air) for 7 min, dry N_2_ for 5 min, and dry fuels for 5 min. The purging steps using fuels instead of N_2_ were conducted to capture reference images during imaging and are discussed in the “Experimental conditions” section.

To determine the effective resistance (also referred to as impedance) of the cell, EIS was performed at each angle by drawing an alternating current perturbed by an amplitude of 10 % of the constant current value. EIS was performed over frequencies ranging from 10 kHz to 0.1 Hz (i.e. starting at 10 kHz and decreasing to 0.1 Hz). For each decade (e.g. between 10  and 1 kHz), 10 logarithmically spaced frequencies were applied and impedance values were obtained at each frequency for a total of 60 measurements per current density. DRT analysis was used to deconvolute the resultant EIS spectra into specific sources of cell resistance or losses and is discussed in detail in the “Dimensionless numbers” section.

### Quantification of liquid water

#### Imaging set-up

To capture the effects of gravity on liquid water accumulation in the fuel cell, *operando* synchrotron X-ray radiography was performed at the Biomedical Imaging and Therapy Facility Insertion Device (BMIT-ID) beamline at the Canadian Light Source (CLS) Synchrotron (Saskatoon, SK)^55^. To spatially resolve liquid water distributions between the inlet and the outlet of the cell, the fuel cell was mounted on the positioning fixture (Fig. 1a) such that the X-ray beam was perpendicular to the length of the cell and path of the flow fields (Fig. 1c and d).

**Fig. 2:**
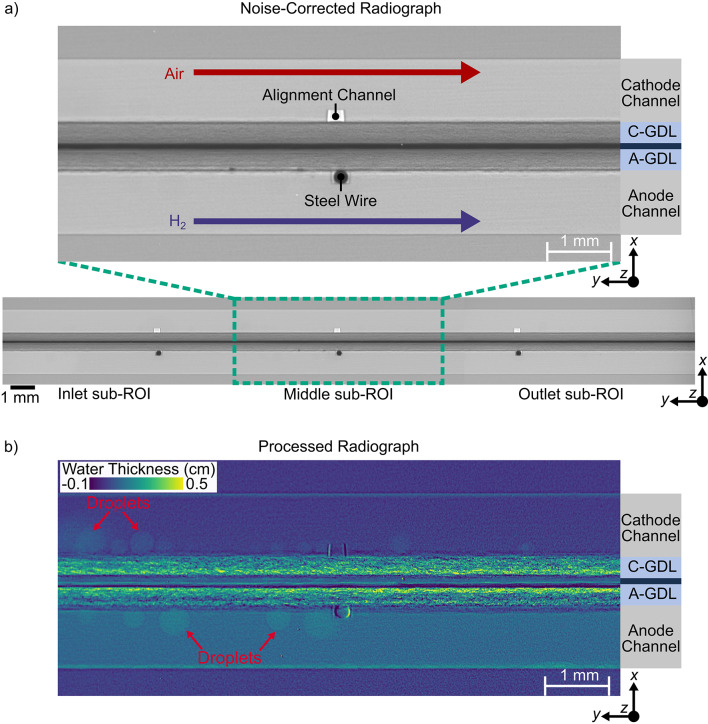
**a** Dark, Flat, and Intensity corrected radiograph of the middle sub-ROI of the fuel cell assembly at the 180° angle, and **b** Resulting radiograph after reference subtraction and application of the Beer-Lambert law to obtain water thickness values in the entire assembly. A high amount of water can be observed in both the anode and cathode GDLs. Water droplets (annotated in red) can also be clearly seen forming from the GDL into the channels of both anode and cathode. Additionally, the entire anode is brighter than the cathode channel, indicating that at least one anode channel was completely full of liquid water during operation.

The fuel cell was aligned manually in the *x* and *y* rotation axes (Fig. 1a) using empty alignment channels in the cathode and steel wires embedded in alignment channels in the anode (see Fig. 2a). To provide adequate contrast between liquid water and fuel cell components, a beam energy of 30 keV was selected. A 0.8 mm Al filter was employed to reduce beam hardening. To convert the attenuated beam into visible light, an AA40 scintillator (CRYTUR spol. S r. o.) was employed. An ORCA-Flash4.0 camera (Hamamatsu Photonics K.K.) then converted the visible light into digital images. This scintillator and camera pairing resulted in a field of view (FOV) of 9.75 mm × 9.75 mm with a pixel size of 6.5 μm. The region of interest (ROI) in the fuel cell was 3.5 mm in width to capture the combined flow field and MEA thickness, and 29 mm in length (obstructive clamping bolts made capturing the full 40 mm length of the active area impossible—see Fig. 1d).

The full ROI of the fuel cell was ~ 3× longer than the field of view available from the imaging setup. Therefore, to capture the full ROI, the fuel cell was imaged at three locations to capture 3 sub-ROIs of the fuel cell assembly (denoted in Figs. 1d and 2a): the inlet sub-ROI (adjacent to the flow field inlet), the middle sub-ROI, and the outlet sub-ROI (adjacent to the flow field outlet). In Fig. 1d, the location of the fuel cell is such that the attenuated X-rays passing through the middle sub-ROI are captured by the scintillator and camera. To capture each sub-ROI, the fuel cell was moved by remotely controlling the imaging stage in the *x* and *y* directions during the experiments. During each constant current density step, the imaging stage was first located to capture the inlet sub-ROI (capturing 1/3 of the full ROI). Based on our previous studies, we assume water transport in the cell is at steady state after approximately 10 minutes^20,35,37,56^; therefore, we held the constant current at the inlet sub-ROI conservatively for 15 min. While continuing to hold constant current, the subsequent sub-ROIs were only imaged for 2.5 min each, since we assumed we had already reached steady state conditions. Similarly, during cell purging between tests, the inlet sub-ROI was imaged for 5 min for wet purging and the middle and outlet sub-ROIs were imaged for 1 min each. For dry purging, the inlet sub-ROI was imaged for 3 min before imaging the middle and outlet sub-ROIs for 1 min each. At the end of each experiment, the beam was turned off to capture dark-field images for approximately 30 s. After capturing dark-field images, the beam was turned back on and the fuel cell was moved out of the field of view to capture 100 frames of flat-field images. All images were captured at 2.5 frames per second (exposure time of 0.35 s with a 0.05 s field delay).

#### Image processing

All image processing described in this section was performed using ImageJ^57^ (National Institutes of Health, Maryland, USA) functions and custom in-house ImageJ macros. Each imaging experiment corresponded to each cell angle in Fig. 1b, and resulted in three stacks of integrated through-plane radiographs (herein referred to as the experimental stacks) for each of the three sub-ROIs defined previously. Dark-field images were averaged and subtracted from all images in the three experimental stacks as well as from the averaged flat-field images. After dark-field correction, beam noise was removed by dividing the dark-corrected experimental stacks by the average intensity of the flat-field images (obtained prior to each experiment). This division resulted in images with intensities normalized between 0 and 1, and these values were less than 1 since the average flat field intensity is much larger than any of the intensities measured within the experimental stacks. The flat field images have significantly higher intensities since they are only attenuated by the ambient air in front of the detector. However, working with intensity values < 1 leads to unphysical results after subsequent calculations (e.g. natural logarithm). Therefore, to work with intensity values > 1, the normalized stack was multiplied by the average flat-field intensity. Dark-field and flat-field corrections are shown together in the following relation:

1 $${I}^{{\prime}}(x,y,n)=\overline{{I}_{flat}}\frac{{I}\left(x,y,n\right)-{I}_{dark}(x,y)}{{I}_{flat}\left(x,\:y\right)-{I}_{dark}(x,y)}$$

where, $\:{I}^{{\prime\:}}$ is the dark and flat-corrected intensity of the experimental stack, $\:{I}$ is the intensity of all radiographs in the experimental stack, $\:{I}_{dark}$ is the intensity of the averaged dark-field image, $\:{I}_{flat}$ is the average intensity of 100 flat-field images, and $\:\overline{{I}_{flat}}$ is a single value representing the spatially averaged intensity value of $\:{I}_{flat}(x,y)$. The coordinate variable, *n* represents the frame number at each time, *t*, based on the frame rate specified previously.

Next, to correct for any intensity changes during the experiments due to slight fluctuations in beam energy^55^, the intensity of each image in the dark and flat-corrected experimental stacks was normalized to the intensity of the first image in the stack. This intensity correction was performed by determining the relative intensity difference between the first image in the stack and each subsequent image in an area of the image not expected to change over the course of the experiment (i.e. a solid portion of the graphite flow field plate):

2 $$\:{I}^{{\prime}{\prime}}(x,y,{n}_{k})={I}^{{\prime}}(x,y,{n}_{k})\frac{\frac{1}{{N}_{x}\cdot{N}_{y}}{\sum}_{i=1}^{{N}_{x}}{\sum}_{j=1}^{{N}_{y}}{I}^{{\prime}}({x}_{i},{y}_{j},{n}_{o})}{\frac{1}{{N}_{x}\cdot{N}_{y}}{\sum}_{i=1}^{{N}_{x}}{\sum}_{j=1}^{{N}_{y}}{I}^{{\prime}}({x}_{i},{y}_{j},{n}_{k})}$$

where, $\:{I}^{{\prime}{\prime}}$ is the now fully corrected intensity of the experimental stack, $\:{N}_{x}$ and $\:{N}_{y}$ are the number of pixels in the *x* and *y* directions used for the intensity correction, (${x}_{i},{y}_{j}$) is the coordinate of pixel, ($i,j$), *n*_*k*_ is the frame number being corrected, and ${n}_{o}$ is the frame number of the radiograph used as the reference (the first image in the stack in our case).

To create a stack of images containing the full ROI of the cell, the three fully corrected sub-ROIs were merged and aligned with one another. Since each sub-ROI was imaged for different lengths of time, each sub-ROI stack was first trimmed to the last 150 frames (60 s) of each current density before merging each sub-ROI stack with one another. A sample merged image is shown in Fig. 2a. Alignment of the sub-ROIs was achieved through a combination of manual alignment and the ImageJ plugin, Template Matching^58^ which aligns stacks of images based on a user-selected region of the image that remains constant throughout the stack (i.e. the alignment wires/channels). After these operations, it was assumed that changes in intensity from the beginning to the end of each combined experimental stack were due to liquid water accumulation from the fuel cell reaction and condensation from humidified gases. To quantify the liquid water accumulation in the cell, the Beer-Lambert Law^35,59,60^ was employed to correlate the intensity of the water to its thickness in the in-plane direction (*z*-axis in Fig. 2b):

3 $${b}_{w}(x,y,{n}_{k})=\frac{1}{{\mu}_{w}}\mathrm{ln}\left(\frac{{I}_{ref}(x,y,{n}_{k})}{{I}_{op}(x,y)}\right)$$

where $\:{b}_{w}$ is liquid water thickness [cm] at each spatio-temporal position, (*x*,*y*,*n*) in the image stack, $\:{\mu\:}_{w}$ is the liquid water attenuation coefficient [cm^− 1^], which was experimentally determined using the methods of Ge et al.^61^ to be 3.222 × 10^− 1^ ± 1.666 × 10^− 5^ cm^− 1^, $\:{I}_{op}$ is the intensities at each position, (*x*,*y*), of the combined experimental image stack at each frame, *k*, during the experiment, and $\:{I}_{ref}$ is the intensity at each position, (*x*,*y*), captured during OCV and averaged over 150 frames (60 s). A sample processed image is shown in Fig. 2b, where water thickness in [cm] is visible in the channels and GDLs of both the anode and cathode.

#### Calculation of water thickness profiles

At each current density, water saturation profiles were calculated for both the anode and cathode across the GDL and channels separately in the flow direction (*x*-direction in Fig. 2b) and in the through-plane direction (*y*-direction in Fig. 2b). To do so, frames of each current density step in the combined processed stack were first averaged, creating a sub-stack of 2D arrays corresponding to average water thickness at each constant current density step in the full ROI of the cell. To create 1D water thickness profiles, we employed spatio-temporal averaging described by:

Flow direction:

4 $$\overline{{b}_{w}}\left(x\right)=\frac{1}{N\cdot{N}_{y}}{\sum}_{k=1}^{N}{\sum}_{j=1}^{{N}_{y}}{b}_{w}(x,{y}_{j},{n}_{k})$$

Through-plane direction:

5 $$\overline{{b}_{w}}\left(y\right)=\frac{1}{N\cdot{N}_{x}}{\sum}_{k=1}^{N}{\sum}_{i=1}^{{N}_{x}}{b}_{w}({x}_{i},y,{n}_{k})$$

where, for the flow direction profiles, water thickness was averaged along the through-plane direction, *y*, at each flow direction position, *x*. For the through-plane profiles, the water thickness at each flow direction position, *x*, was averaged along the through-plane direction, *y*. For both equations, $\overline{{b}_{w}}$ is the average water thickness [cm] at each flow direction or through-plane position, $N$ is the number of frames averaged (150), $\:{N}_{x}$ and $\:{N}_{y}$ are the number of pixels averaged at each flow direction or through-plane position, respectively. Separate water thickness profiles in each direction were created for the GDL and the channels.

To determine the uncertainty in calculated liquid water thickness due to optical instrumentation noise (from the camera, scintillator, and beam) in addition to the spatial and temporal variance in water thickness, we employed the methods of Chevalier et al.^60^. Uncertainties in water thickness found in this study were $\le$ 0.005 cm, corresponding to less than 1 % of the average saturation intensity in each test, which agrees with Chevalier et al.^60^.

#### Calculation of GDL saturation profiles

To determine the liquid water fraction in the channels and GDLs, water thickness calculated in Eqs. 4 and 5 and corresponding uncertainty were divided by the total thickness of the channels and the GDL, respectively:

6 $$VF\left(\xi\right)=\frac{{b}_{w}\left(\xi\right)}{L}\:\:\:\:\:\:\xi\in\{x,y\}$$

where $\xi$ is a placeholder for either the $x$ or $y$ direction, $VF\left(\xi\right)$ is the volume fraction profile of water in the flow direction for $\xi=x$, and in the through-plane direction if $\xi=y$, and *L* is the in-plane thickness in [cm] of the channels (0.4 cm) or GDL (0.8 cm).

To calculate the saturation profiles of the porous GDL based on the liquid water volume fraction and GDL porosity, we first determined 1D porosity profiles of the GDL along the through-plane and flow directions. These porosity profiles were determined via ex-situ micro computed tomography (µCT) (CT Mini, ProCon X-ray GmbH, Germany). During µCT imaging, the GDL was compressed using a custom sample holder with land-channel geometry. Additionally, the same PTFE gaskets used in the *operando* experiments were employed in the ex-situ characterization to emulate the compression experienced by the GDL in the operating fuel cell. The µCT was conducted using an X-ray voltage of 35 keV, 200 µA, an exposure time of 0.721 s, frame averaging of 3, resolution of 3.00 μm px^− 1^, and 2600 projections over 360-degree rotation. All acquired projections were reconstructed using VGSTUDIO MAX (Volume Graphics GmbH, Heidelberg, Germany) and segmented into GDL substrate, MPL, and void space via the Trainable Weka Segmentation plugin^62^ in ImageJ. The resolution of the CT scan was not adequate to resolve the sub-micron pores of the MPL; therefore, we assumed the solid parts of the MPL had a porosity of 0.5 based on previous studies that resolved MPL porosities in similar GDL materials^63,64^. The porosity of the GDL was calculated at each flow direction position, *x*, and through-plane position, *y* via the following relation:

7 $$\epsilon\left(\xi\right)=\frac{Void\:Volume\left({\upxi}\right)}{MPL\:Volume\left(\xi\right)+GDL\:Volume\left(\xi\right)+Void\:Volume\left(\xi\:\right)}\:\:\:\:\:\xi\in\{x,y\}$$

where $\epsilon\left(\xi\right)$ is the porosity profile of the GDL along the flow direction position for $\xi=x$ and along the through-plane position, for $\xi=y$. For the purposes of saturation calculation, it was assumed that the porosity profiles determined via µCT were the same as the porosity profiles of the GDL used for the *operando* radiography. The GDL saturation profile was calculated from the GDL liquid volume fraction profiles via:

8 $$s\left(\xi\right)=\frac{VF\left(\xi\right)}{\epsilon\left(\xi\right)}\:\:\:\:\:\:\xi\in\{x,y\}$$

where $s\left(\xi\right)$ is the saturation profile of the GDL along the flow direction for $\xi=x$ and along the through-plane direction, for $\xi=y$.

### Dimensionless numbers

Since this study is concerned with the effect of gravity on transport in the fuel cell, the Bond number was calculated in both the channels and GDL using:

9 $$Bo=\frac{{\Delta}\rho{g}{R}^{2}}{\sigma}$$

where, ${\Delta\:}\rho$ is the difference between the density of air and water (982 kg m^− 3^) at the fuel cell operating temperature (60 °C), $g$ is the acceleration due to gravity (9.81 m s^− 2^), $R$ is the characteristic length of the channel or GDL, and $\sigma$ is the surface tension of water in air at 60 °C (0.0662 N m^−1^). The characteristic length, *R*, was the hydraulic diameter of the channels (1 mm), and the average pore diameter (18.5 μm) of the GDL for the Bond number of the channels and GDL, respectively. The average pore diameter of the GDL was considered the inscribed diameter calculated via the custom watershed algorithm and pore network extraction functions developed by Gostick^65^ on the µCT image of the compressed GDL. A pore size distribution of the GDL is shown in Figure S1.

To determine the relative contributions of the inertial forces of both reactant gases (hydrogen and air) to the gravitational force resisting liquid water removal from the channels, we also calculated a modified Froude number defined as follows^66^:

10 $$Fr{\prime}=\frac{{\rho}_{g}{U}^{2}}{{\rho}_{l}gL}$$

where, ${\rho}_{g}$ and ${\rho}_{l}$ are the densities [kg m^− 3^] of the gas and liquid phases, respectively, $U$ is the velocity of the gas phase in a single channel (2.08 m s^− 1^), and $\:L$ is the characteristic length scale [m] (droplet diameter in this case). At the fuel cell operating temperature (60 °C), the density of hydrogen is 0.0899 kg m^− 3^, the density of air is 1.06 kg m^− 3^, and the density of water is 983 kg m^− 3^.

### Distribution of relaxation times (DRT)

To determine the effect of gravity on specific sources of electrochemical losses in the fuel cell, the distribution of relaxation times (DRT) method was employed for the EIS spectra captured at each cell angle. The EIS spectra at 0.6 A cm^− 2^ were specifically used as this was the highest current density reached by all cell angles. All analysis on EIS spectra in this study was performed using RelaxIS3 (rhd instruments GmbH & Co. KG, Darmstadt, Germany). To reduce noise in the raw EIS spectra, a Savitzky-Golay filter^67^ was applied with a smoothing factor of 0.1. Furthermore, to certify the validity of the EIS data, a Kramers-Kronig Test (KKT) filter was performed to ensure all remaining EIS data points had a relative error of < 1 %.

The DRT method involves modelling the impedance spectra of the fuel cell as the integration of an infinite series of resistive-capacitive circuits and is inherently an ill-posed problem as the integration does not have a unique solution^68^. To convert the integration into a well-posed problem, a regularization parameter, $\:\lambda\:$ is commonly employed^68^. For this study, a value of $\lambda$ = 1E-5 was selected as the optimal regularization parameter value to minimize changes in the sum of squares residual (SSR) between the modelled DRT spectra and the raw EIS spectra of each test conducted in this study (Figure S2). The same regularization parameter was used for DRT analysis of all spectra in this study. Peak assignment and DRT calibration experiments are outlined in the following subsection.

#### DRT peak assignment via calibration experiments

In this study, we determined the significance of mass transport losses in the MEA and flow field channels. To identify the DRT peaks associated with reactant diffusion resistance and mass transport resistance, we followed the methods of Weiss et al.^48^ and conducted calibration experiments on our fuel cell using oxygen (herein referred to as the H_2_/O_2_ tests) as the oxidant rather than air (H_2_/Air tests). This method provides abundant oxygen gas to the cathode to eliminate mass transport losses associated with oxygen diffusion. Using oxygen instead of air on the cathode can also reduce mass transport losses on the anode. For example, the use of oxygen instead of air eliminates the risk of N_2_ crossover to the anode, which can lead to hydrogen starvation – especially when significant liquid water accumulates at the anode^68,69^. We used 70 % O_2_ mixed with 30 % N_2_ (rather than 100 % O_2_ used by Weiss et al.^48^, which was adequate in eliminating mass transport losses under our experimental conditions. All other conditions were the same as those described in the “Experimental conditions” section. Due to the abundant supply of oxygen during H_2_/O_2_ tests, we did not observe significant changes in performance at different cell angles.

**Fig. 3:**
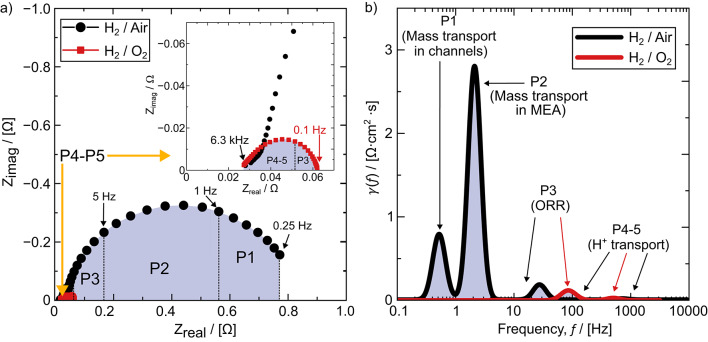
**a** Sample impedance spectra for H2/Air and H2/O2 fuel cell operation and **b** resultant distribution of relaxation times (DRT) multiplied by the active area with impedance peaks labelled with their related sources in the fuel cell.

Sample EIS spectra of the experiments using H_2_/O_2_ and H_2_/Air at 0.6 A cm^− 2^ are shown in Fig. 3a, and the corresponding DRT response, $\gamma\left(f\right)$, is shown in Fig. 3b, where the resistance peaks are labelled as P1–P5. Only P3, P4, and P5 were present in both H_2_/O_2_ and H_2_/Air DRT responses, indicating that these peaks do not represent mass transport losses. Additionally, the frequency and magnitude of P3 and P4–P5 agreed with values in the literature associated with the ORR and proton transport resistance in the catalyst layer, respectively^41,48,49^. Therefore, for this study, the peaks at frequencies > 10 Hz were attributed to the ORR (P3) and proton transport losses in the CL (P4 and P5). It should be noted that P3 was centred at a significantly higher frequency when oxygen was used instead of air. We attribute this shift in frequency to the abundance of oxygen available in the H_2_/O_2_ experiments, which led to a faster rate of reaction for the ORR. There is also some small deviation in the frequencies of P4 and P5, which is expected between different experiments^10^. The remaining two peaks at low frequencies (P1, P2) only present in the H_2_/Air DRT were attributed to mass transport losses in the cell assembly. Based on previous models of EIS spectra and DRT in low temperature PEM fuel cells from Kulikovsky^45,46^, we attribute the left-most peak at the lowest frequency to mass transport losses in the flow field channels. Additionally, this low frequency peak strongly corresponded with observations of water slugs in the channels (discussed further in the results section), further justifying this attribution.

## Results

### Improved electrochemical cell performance with gravity assisted flow

**Fig. 4:**
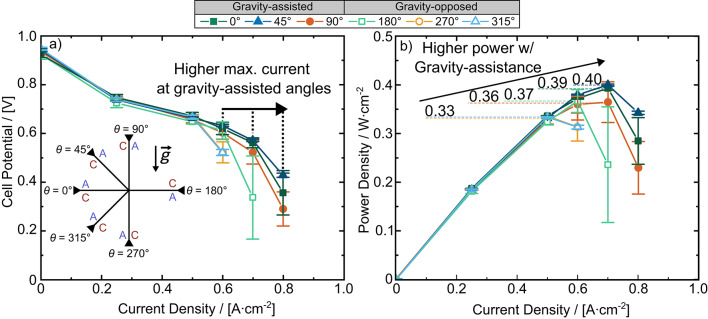
Average **a** polarization curve and **b** power density curve for each angle. Error bars represent one standard deviation between cell potential recorded during the last minute of all tests.

Electrochemical performance testing revealed significant improvements to fuel cell performance when gravity assisted the flow of reactants and product water towards the cell outlet (Fig. 4). Specifically, when the fuel cell was positioned at gravity-assisted angles (45° and 90°), a maximum current density of 0.8 A cm^− 2^ was reached. At gravity-opposed angles (270° and 315°) the fuel cell only reached a maximum current density of 0.6 A cm^− 2^, after which the cell failed due to mass transport limitations. The peak power densities of the gravity-assisted angles were up to 21.2 % higher than gravity-opposed angles. Specifically, the peak power density at both 90° and 45° was only 0.33 W cm^− 2^, while at 270° and 315° the fuel cell achieved peak power densities of 0.36 W cm^− 2^ and 0.40 W cm^− 2^, respectively (see Fig. 4b). Furthermore, the peak power densities of gravity-assisted angles were reached at a current density of 0.7 A cm^− 2^, whereas gravity opposed angles reached their peak power densities at 0.5 A cm^− 2^. We attribute the improved mass transport behaviour to the gravity-assisted angles that enhanced liquid water removal from the flow field channels. The enhanced water removal at gravity-assisted angles from the channels led to more effective advective water removal from the GDL and improved gas reactant transport to the CL.

Surprisingly, a distinct polarization difference was observed between the two horizontal positions (0° and 180°), which one might have assumed to be gravity neutral. At 0°, where the cathode was placed below the anode, the fuel cell reached the same maximum current density (0.8 A cm^− 2^) as the gravity assisted angles (Fig. 4a). In contrast, at 180°, the angle in which the cathode is positioned above the anode, the fuel cell achieved a peak power density 5.12 % lower than at 0° (0.37 W cm^− 2^ vs. 0.39 W cm^− 2^) and only reached a current density of 0.7 A cm^− 2^ (Fig. 4b). The 180° angle is henceforth considered a gravity-opposed angle, and the 0° angle is considered gravity-assisted. Although the performance improvement at 0° is marginal, we attribute the superior performance to the enhanced removal of liquid water from the GDLs and channels. To elucidate further, we next apply *operando* synchrotron X-ray radiography to quantify liquid water accumulation in the flow field channels and GDL at each angle. For the remainder of this paper, all water accumulation and EIS measurements are presented at the highest current density that was reached for all fuel cell angles (0.6 A cm^− 2^).

To determine whether statistical significance of cell potential was observed between the various cell angles, an analysis of variance (ANOVA) was performed for all fuel cell angles and each current density step shown in Fig. 4a. The results of the ANOVA analysis are shown in Table S1 and indicate extremely significant differences between the datasets (i.e. between each tested angle). This indicates that the cell potentials at each angle are due to the cell angle and not attributed to variance between fuel cell assemblies. While the ANOVA analysis does not change the outcomes of this study, it is important to note that ANOVA provides a comparison between all angles and that for some angles such as 45° and 90° or 270° and 315°, the mean cell potentials and standard deviations at each current step are nearly identical indicating that the performance of these angles are similar to each other.

### Flow orientation affects liquid water removal from gas diffusion layers

**Fig. 5:**
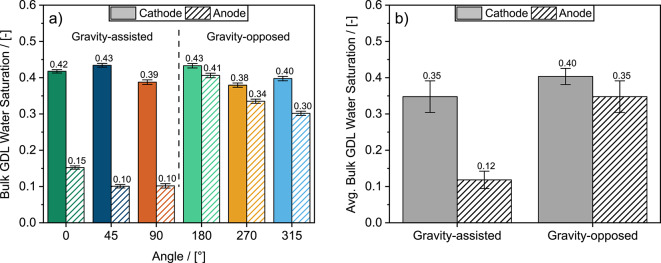
**a** Bulk water saturations at 0.6 A cm^− 2^ for the anode and cathode GDLs at each angle and **b** Averaged bulk water saturation values from a) for all gravity-assisted and gravity-opposed angles. Error bars in **a** represent uncertainty associated with temporal and spatially statistical variance in water thickness as well as from noise and inherent uncertainty from the optical instrumentation (scintillator, camera, and beam energy). Error bars in **b** represent one standard deviation between the angles.

Through *operando* synchrotron X-ray radiography, we found the difference in bulk anode GDL water saturation between gravity-assisted (90°, 45°, 0°) and gravity-opposed (270°, 315°, 180°) angles was significant compared to the relatively constant cathode GDL water saturation across all angles (see Fig. 5a). In contrast to the cathode GDL, the average bulk water saturation (Fig. 5b) in the anode GDL was significantly higher (≥ 0.30) for gravity-opposed angles compared to gravity-assisted angles (≤ 0.15), demonstrating how gravity had a more significant effect on liquid water accumulation in the anode compared to the cathode. These results agree with the operando imaging findings of Spendelow et al.^33^ who also concluded that gravity has a more significant effect on liquid water in the anode compared to the cathode. Additionally, consistent with Spendelow et al.^33^, we observed more significant water accumulation with the cathode above the anode (180°) instead of below it (0°).

**Fig. 6:**
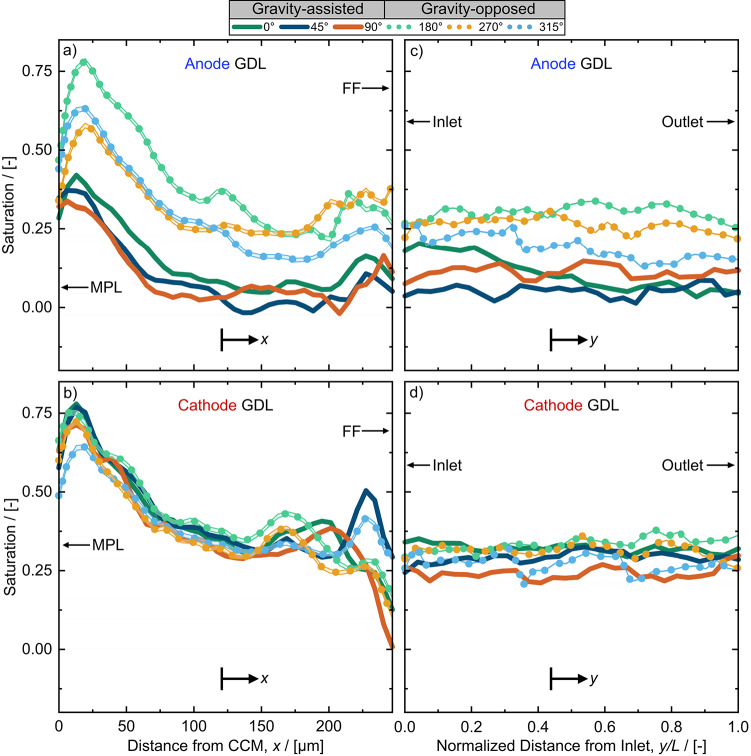
Water saturation profiles in the **a** anode and **b** cathode GDLs from the MPL interface to the flow field (FF) interface (through-plane, *x*) at 0.6 A cm^− 2^ and from the inlet to outlet of the fuel cell (flow direction, y) in the **c** anode and **d** cathode GDLs. In **c** and **d** the distance from the inlet to the outlet of the channels, *y*, is normalized by the length, L, of the merged sub-ROIs. Uncertainty of water thickness is represented by a shaded line region between data points but are not easily visible due to their extremely low values (typically < 1 % of the saturation). Negative values are attributed to insignificant material movement and noise inherent to the optical equipment, both of which are not considered physical values of water thickness. To avoid misrepresentation of more significant negative values as saturation, the MPL and 5 pixels of the GDL near the FF interface were omitted from this analysis.

We also reveal significantly higher liquid water saturations in the anode GDL in gravity opposed angles compared to gravity-assisted angles via the through-plane water saturation profiles (see Fig. 6a). This difference in liquid water saturation is most significant near the MPL interface where the obstruction to reactant delivery to the CL is most impactful. Severe water flooding in the anode GDL near the CL may have caused hydrogen starvation, leading to significant polarization losses and sudden cell failure in the mass transport region of the polarization curves that we observed for gravity-opposed orientations (Figs. 4 and S2). Conversely, liquid water saturation in the cathode GDL generally remained consistently high at all angles with only small differences observed between gravity-opposed and gravity-assisted angles near the GDL – flow field interface. The large quantities of liquid water at the cathode for every angle likely also contributed to the sudden cell failure (Figure S3) observed for gravity-opposed angles that additionally experienced severe liquid water flooding in the anode compared to gravity-assisted angles.

The local saturation of water in the GDL was insensitive to the distance from the inlet of the fuel cell (see Fig. 6c and d). The insensitivity of the GDL water saturation to the distance from the inlet is attributed to the relatively low Bond number in the GDL (4.98 × 10^− 5^, calculated using Eq. 9), indicating the dominance of capillary pressure over gravitational forces in the GDL. In contrast, the impact of gravity was likely more significant in the cathode and anode channels compared to within the GDLs due to the channel Bond number of 0.13 (based on 1 mm x 1 mm channels).

The uncertainty values presented as error bars in Fig. 5 and shaded regions between points in Fig. 6 represent temporal and spatially statistical variance in water thickness and inherent noise from the optical instrumentation (i.e. scintillator, camera, and beam energy) discussed in the “Calculation of water thickness profiles” section. The values of uncertainty were on average < 1% of the liquid water saturation values and are therefore difficult to discern in the liquid water profiles of Fig. 6. Similar behaviour was observed for values of liquid water in the channels presented in the following sub-section.

### Gravity significantly impedes water removal from flow field channels

**Fig. 7:**
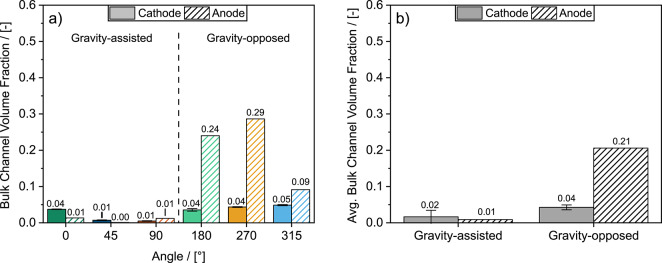
**a** Bulk water saturations at 0.6 A cm^− 2^ for the anode and cathode channels at each angle and **b** Averaged bulk water saturation values from **a** for all gravity-assisted and gravity-opposed angles. Error bars in **a** represent uncertainty associated with temporal and spatially statistical variance in water thickness as well as from noise and inherent uncertainty from the optical instrumentation (scintillator, camera, and beam energy). Error bars in **b** represent one standard deviation between the angles.

Liquid water accumulated significantly in the anode channels and moderately in the cathode channels at gravity-opposed angles (see Fig. 7). At these gravity-opposed angles, the bulk anode channel water accumulation was substantial, with an average bulk water fraction of 0.21. The reactant hydrogen was not sufficiently effective at overcoming the shear, capillary, and especially the gravitational forces that resist droplet removal in the channels. We therefore attribute this ineffective water removal in the anode channels to the lower inertial force of hydrogen relative to air, compared to the force of gravity acting on the liquid water to resist removal from the channels. This claim is supported by our calculations of a modified Froude number ($Fr^{\prime}$) defined by Eq. 10. For our system, ${Fr}^{{\prime}}$= 0.88 for air and 0.075 for hydrogen, assuming a water droplet diameter of 0.5 mm. The $Fr{\prime}$ for hydrogen is an order of magnitude smaller than that of air, demonstrating that in the anode, the force of gravity is significantly more relevant to the removal of liquid water. Furthermore, the lower dynamic viscosity of hydrogen (9.62 μPa s) compared to air (20.0 μPa s), would have resulted in a lower pressure drop across the channels, furthering limiting water removal from the anode. As a result of hindered water removal, large water droplets that eventually developed into slugs were observed in the anode channels for all gravity-opposed angles (example shown in Supplementary video 1). Compared to the significant water accumulation in the anode channels, the cathode channel water accumulation at gravity-opposed angles was far less, with an average bulk water fraction of 0.04 (see Fig. 7b). Still, more liquid water was observed in the cathode channels when gravity was opposed, exhibited by a difference of 0.02 bulk water fraction between gravity-opposed and gravity-assisted angles.

**Fig. 8:**
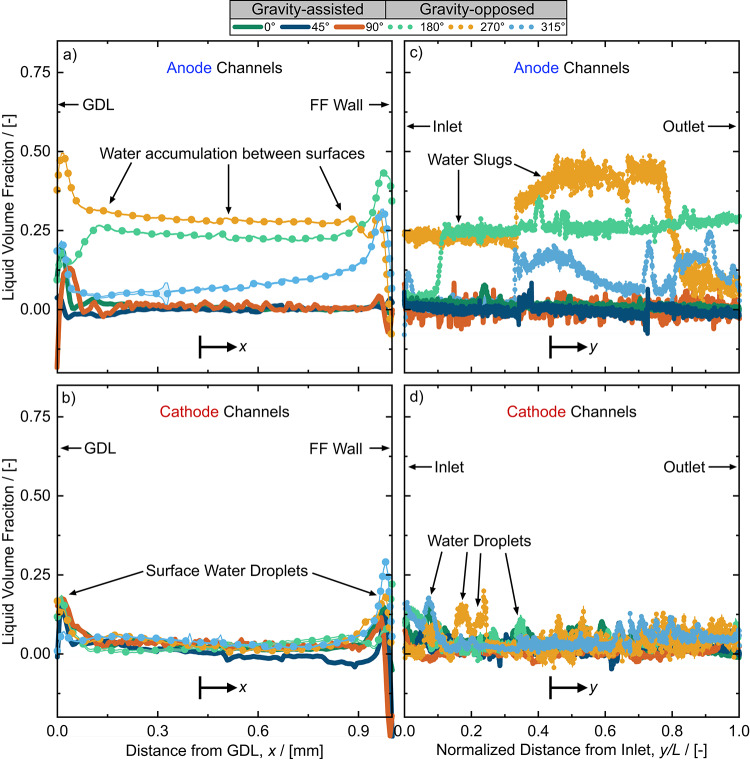
Water saturation profiles in the **a** anode and **b** cathode channels from the GDL interface to the wall of the flow field channel interface (through-plane, *x*) at 0.6 A cm^− 2^ and from the inlet to outlet of the fuel cell (flow direction, *y*) in the **c** anode and **d** cathode channels. In **c** and **d** the distance from the inlet to the outlet of the channels, *y*, is normalized by the length, *L*, of the merged sub-ROIs. Uncertainties of water thickness are represented by a shaded line region between data points but are not very visible due to their extremely low values (typically < 1 % of the saturation). Negative values are attributed to negligible material movement and noise inherent to the optical equipment, both of which are not considered physical values of water thickness.

We also quantified the liquid water content in the channels in two directions: (1) the through-plane position from the GDL interface to the flow field wall (Fig. 8a, b), and (2) the flow direction from the channel inlet to the outlet (Fig. 8c, d). The through-plane liquid water profile in the anode (Fig. 8a) shows a constant presence of liquid water between the GDL/channel interface and channel/flow field wall interface at gravity-opposed angles and some moderate accumulation at the interfaces for gravity-assisted angles. When reactant flow was gravity-opposed, the added resistance to droplet removal facilitated water droplet growth and coalescence in the anode channels (as observed in Supplementary video 1) resulting in the significant water accumulation observed in Fig. 8a. For gravity-assisted angles, the droplets were removed before significant coalescence occurred, owing to the assistance of gravity. Furthermore, droplet coalescence results in larger droplets with higher masses, increasing the contribution of gravity towards droplet removal at gravity-assisted angles and resistance to droplet removal at gravity-opposed angles. In the cathode channels, (see Fig. 8b) water was only present at the GDL/channel interface (droplets emerging from the GDL) and at the channel/flow field wall interface (condensation droplets). At these two interfaces, only very minor increases in water fraction were observed in the cathode channels for gravity-opposed angles compared to gravity-assisted angles.

Figure 8c and d are particularly useful for illustrating the development of liquid water obstructions along the length of the channels. For example, gravity-opposed angles led to significant liquid volume fractions in the anode channels (up to 25–50 %) for more than half of the anode channel length for gravity-opposed angles (see Fig. 8c). The accumulation of droplets and slugs observed in the anode channel can be observed in Supplementary video 1. Since there were only four channels in the flow field, a liquid volume fraction of 25 % (such as for 270° and 180° in Fig. 8c) implies that one anode channel was fully blocked by a slug of water. The cathode channels were significantly less flooded; however, more numerous and larger droplets are present at the inlet of the cathode channel for gravity-opposed angles compared to gravity-assisted angles (Fig. 8d). This behaviour was evidenced by peaks in liquid volume fraction near the left side of the Fig. 8d (annotated as ‘water droplets’). Droplet accumulation near the flow field inlet at gravity-opposed angles is attributed to the higher resistance to droplet removal due to gravity. Droplet accumulation and full obstructions (such as in the anode) result in local reactant starvation, leading to significant increases in mass transport losses and rapid cell failure, as seen in the performance curves in Figs. 4 and S2. To determine the contribution of mass transport losses to the overall cell resistance, DRT analysis was employed in the following section.

### Distribution of relaxation times unveils the significance of transport resistance through flow field channels

**Fig. 9:**
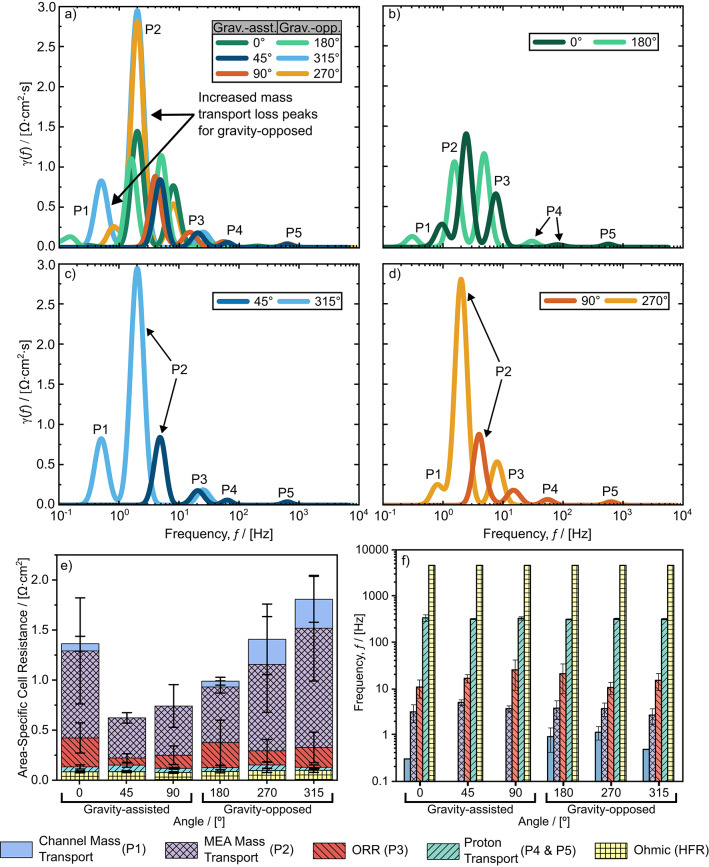
**a** Sample DRT result of each angle at 0.6 A cm^− 2^ using a 1st order Gaussian discretization and *λ* = 1E-5. **b**–**d** Opposite angles shown together for ease of comparison. **e** Component breakdown of each source of cell resistance and **f** corresponding frequency obtained from DRT analysis.

From the calibration experiments presented in the methodology, the DRT peaks for each cell angle were assigned to specific mechanisms of loss in the cell. Selected DRT curves at 0.6 A cm^2^ (from the EIS of one fuel cell assembly) are compared in Fig. 9a–d for each cell angle. At each angle, the area-specific resistance (ASR) was calculated for each loss mechanism by integrating the DRT peaks across their respective frequency ranges. This process was repeated for each fuel cell assembly. The average ASR and average centre frequency of each peak are shown for each cell angle in Fig. 9e and f, respectively.

The largest peak for all cell angles was P2, which is related to mass transport losses in the MEA (Fig. 9a–d). The average ASR of P2 (in Fig. 9e) was lower for gravity-assisted angles, 90° and 45°, compared to the 0° angle and the gravity-opposed angles (180 °, 270°, and 315°). This decrease in MEA mass transport resistance coincides with the superior performance of these gravity-assisted angles at current densities ≥ 0.6 A cm^2^ (Fig. 4). Typically, the contribution to mass transport losses in MEA from the anode has been overlooked within the literature. However, cathode GDL water accumulation was similar for all angles, but anode GDL water accumulation was significantly higher at 0° and gravity-opposed angles compared to 45° and 90° (Figs. 5 and 6). Therefore, we attribute the high ASR of P2 at 0° and gravity-opposed angles to mass transport losses due to diffusion limited transport in the anode. It would have been otherwise challenging to uniquely and simultaneously identify both anode and cathode GDL mass transport losses solely with DRT (since they occur at similar frequencies), but we overcame this challenge by combining DRT with *operando* imaging. This unique combination allowed us to be the first to explicitly identify anode liquid water accumulation to mass transport losses with DRT. Furthermore, we found that the peak at the lowest frequencies in the DRT response (P1), only manifested at angles with significant anode and cathode channel water accumulation (0°, 180°, 270°, and 315°—Fig. 7). Through the combination of DRT and *operando* imaging, we can therefore attribute P1 to mass transport losses in the cathode and anode channels.

The ASR associated with the ORR peaks (P3) was slightly higher for the gravity-opposed angles and the 0° angle, which we attribute to the sluggish delivery of oxygen to the catalyst layer due to the abundance of liquid water in the cathode channels. Since P4 and P5 both represent losses associated with proton transport in the CL, they can be treated as a single peak for simplicity by summing their ASRs to a single value (reported in Fig. 9e). The sum of the ASR of P4 and P5 was similar for all angles. HFR was similar for every cell angle and observed directly from the EIS spectra at 5 kHz for all cell angles.

The centre frequencies of the ORR (P3) and MEA mass transport loss (P2) related peaks were lower for the gravity-opposed angles, 270° and 315° compared to gravity-assisted angles, 90° and 45° (see Fig. 9f). This decreased frequency of MEA mass transport loss peaks at 270° and 315° was attributed to intense liquid water flooding, which resulted in longer reactant diffusion times in the MEA. The ORR was subsequently slowed due to the delayed delivery of oxygen to the reaction sites. Additionally, flooding of the anode likely blocked reactant sites for hydrogen, thereby reducing the number of available protons that contributed to the ORR, slowing down the reaction even further. The centre frequencies of P4 and P5 were averaged to a single value since they are treated as a single peak and were similar for all cell angles.

## Discussion

In conclusion, through a combination of *operando* synchrotron X-ray radiography and DRT analysis, we have demonstrated the significant benefits of gravity to enhance reactant delivery and product removal in a PEM fuel cell to ultimately improve cell performance. Power density increased by 21.2 % at the gravity-assisted angle of 90° compared to the gravity-opposed angle of 270°, where opposing gravity caused higher mass transport losses due to more significant liquid water accumulation in the channels and GDL – especially in the anode due to the small inertial forces of hydrogen. *Operando* imaging revealed a 0.20 higher bulk water fraction in the anode channels and a 0.02 increase in the cathode channels at gravity-opposed angles, leading to increases in GDL water saturation of 0.18 and 0.1, respectively, at gravity-opposed angles. Additionally, by combining DRT analysis and *operando* imaging, we correlated this water accumulation to mass transport losses in the channel, separately from mass transport losses in the MEA. The additional channel mass transport peak (P1) emerged only at gravity-opposed angles and the 0° angle, driven by significant anode and moderate cathode channel flooding.

Although mass transport losses are typically linked to oxygen starvation in the cathode, our observations indicate that the sudden cell failure and poor performance at gravity-opposed angles were additionally due to anode flooding. The anode flooding promotes hydrogen starvation, which increases anode mass transport losses and may additionally hinder proton transport from the anode CL to the membrane. Anode flooding further reduces the available protons for the oxygen reduction reaction. Additionally, hydrogen starvation can lead to carbon corrosion in the CL resulting in loss of active catalyst surface area, further reducing fuel cell performance and material lifetime. Therefore, in addition to decreasing efficiencies, gravity-opposed angles may also be more susceptible to catalyst layer degradation over long-term operation.

The flow field design can also significantly impact the degree to which gravity affects two-phase flow in PEM fuel cells. Serpentine and interdigitated flow field designs may mitigate the effects of gravity on liquid water accumulation due to their inherently higher pressure drop. The most commonly employed flow fields are multi-serpentine designs^70^, which are made up of a series of straight channel sections such as the flow field we employed. Therefore, we recommend operating fuel cells at an angle that keeps the longest straight sections of the flow field at a 45°–90° angle for optimal electrochemical performance and water removal. Furthermore, liquid water flooding in the flow field is highly dependent on humidity and flow rate, and we recommend that both should be tuned to minimize the adverse effects of gravity on liquid water accumulation and electrochemical performance.

This study demonstrates the critical need to consider the effects of gravity and similar body forces on liquid water accumulation in PEM fuel cells, which become especially relevant for mobile applications. In addition to determining the effect of gravity on PEM fuel cell mass transport and performance, this study demonstrates the first use of combined DRT analysis and *operando* imaging. This unique coupling of characterization methods enabled us to be the first to separately relate liquid water accumulation in the GDL and channels to distinct mass transport peaks in the DRT response. Furthermore, we demonstrated that high mass transport losses in the MEA are directly related to high liquid water accumulation in the anode, which is often overlooked as a source of diffusion losses in PEM fuel cells and difficult to distinguish from cathode mass transport losses without imaging.

## Electronic supplementary material

Below is the link to the electronic supplementary material.

Supplementary Material 1.

## Data Availability

A supplementary video has been made available via: https://doi.org/10.5281/zenodo.15016533. Additional data are available on reasonable request from the corresponding author.
