# Supplementary material for: Influence of gravity on water management and mass transport losses in polymer electrolyte membrane fuel cells
Source: Sci Rep. 2025 Nov 11;15:39380. doi: 10.1038/s41598-025-09067-y (PMC12606143; doi:10.1038/s41598-025-09067-y)
Supplement: Supplementary file 1 — Supplementary Material 1. [file 41598_2025_9067_MOESM1_ESM.docx]

**Electronic Supplementary Information**

**Influence of gravity on water management and mass transport losses in polymer electrolyte membrane fuel cells**

Eric A. Chadwick^a,b^, Beste Derebaşı^a^, Volker P. Schulz^b^ and Aimy Bazylak^a*^

^a^Bazylak Group, Department of Mechanical & Industrial Engineering, Faculty of Applied Science and Engineering, University of Toronto, Toronto, Ontario, Canada

^b^Electrochemical Cluster, Department of Mechanical Engineering, Baden-Württemberg Cooperative State University, Mannheim, Baden-Württemberg, Germany

***Corresponding Author:**

A. Bazylak

Mechanical & Industrial Engineering, Faculty of Applied Science & Engineering

University of Toronto

5 King’s College Road Toronto, ON M5S 3G8

**Section S1. Pore size distribution of compressed Toray120 with MPL**

**
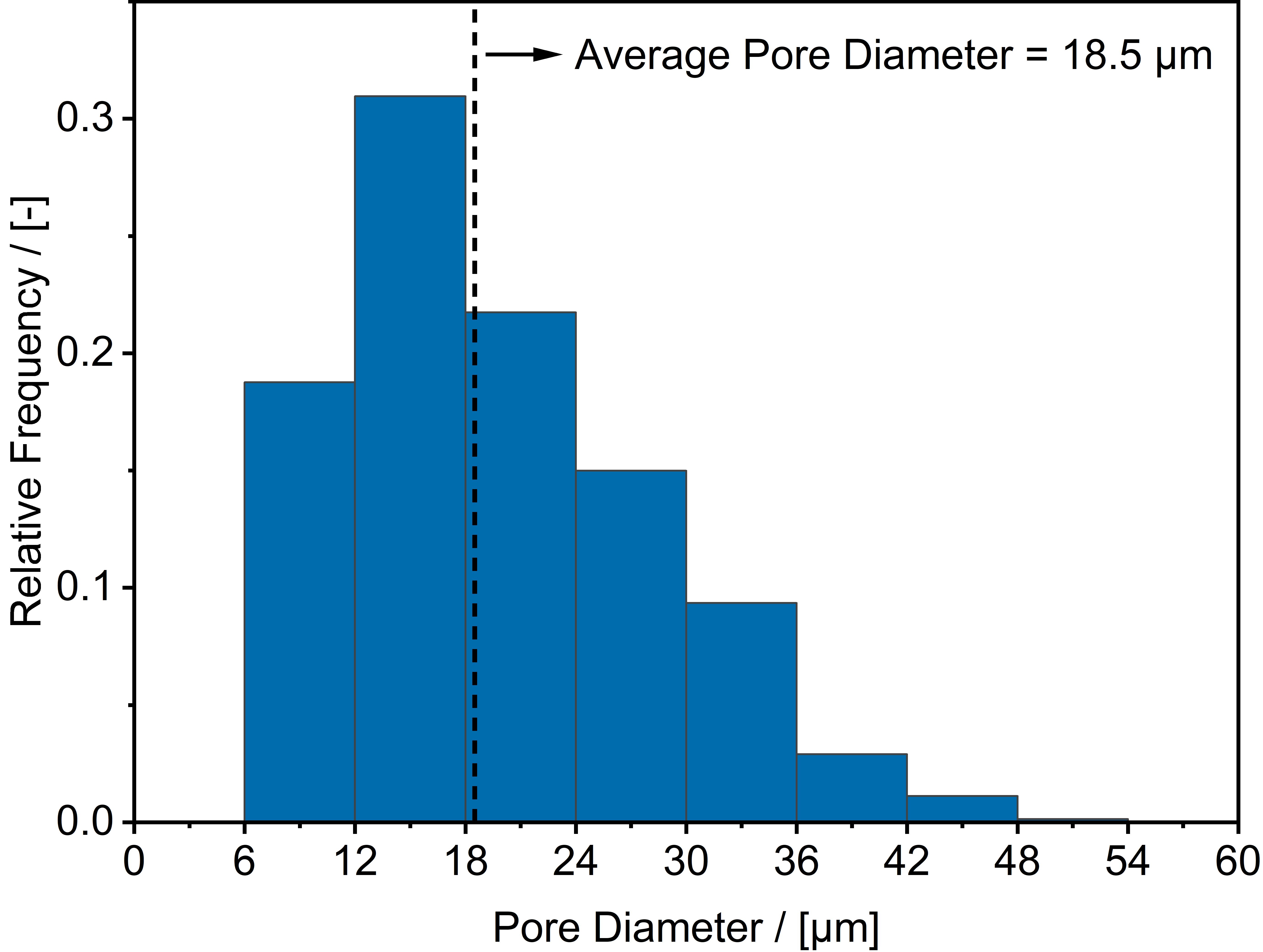
**

Figure S1: Pore size distribution of the compressed Toray120 with MPL GDL used in this study. Pore diameter was determined as the inscribed diameter extracted from the custom watershed and pore network extraction algorithms developed by Gostick^1^

**Section S2. SSR using different regularization parameters for DRT analysis**


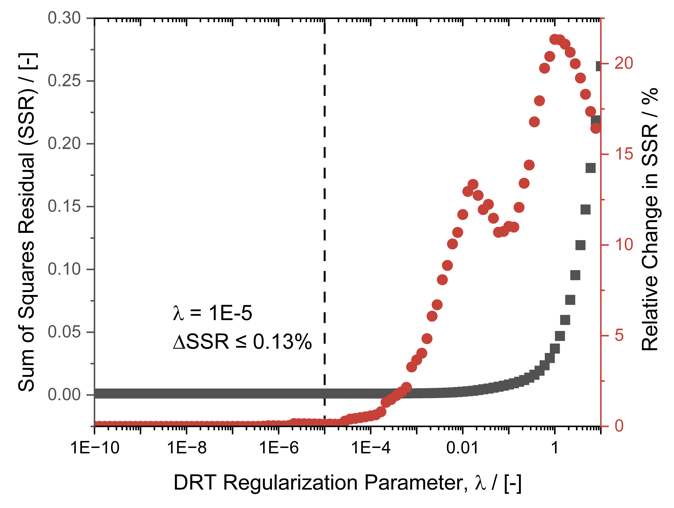


Figure S2: Lambda optimization by comparing lambda values to sum of squares residual (SSR) between the modelled DRT spectra and the raw EIS spectra. Sample data shown is from a 315º angle test. The optimal value of $\lambda$ = 1E-5, was found to be the highest lambda value that produced < 0.1% change in SSR for all EIS spectra used in this study.

**Section S3. ANOVA Analysis of Polarization Data**

**Table S1:** P-values from ANOVA analysis performed in Python for each current density step. ANOVA was performed between all fuel cell angles tested based on sample size (number of averaged cell potential points for each current density), mean cell potential, and standard deviation of cell potential.

| Current Density (A·cm^-2^) | 0 | 0.25 | 0.5 | 0.6 | 0.7^†^ | 0.8 ^‡^ |
| --- | --- | --- | --- | --- | --- | --- |
| P-value | 9.65E-134 | 1.25E-99 | 8.34E-220 | 0.00E+00* | 1.51E-164 | 5.50E-87 |
| *****Value was so low that Python could not display the number of decimal places  ^†^ANOVA performed using data at fuel cell angles 0, 45, 90, and 180 only (others failed before)  ^‡^ANOVA performed using data at fuel cell angles 0, 45, and 90 only (others failed before) | | | | | | |

**Section S4. Sample transient polarization curves at each fuel cell angle.**


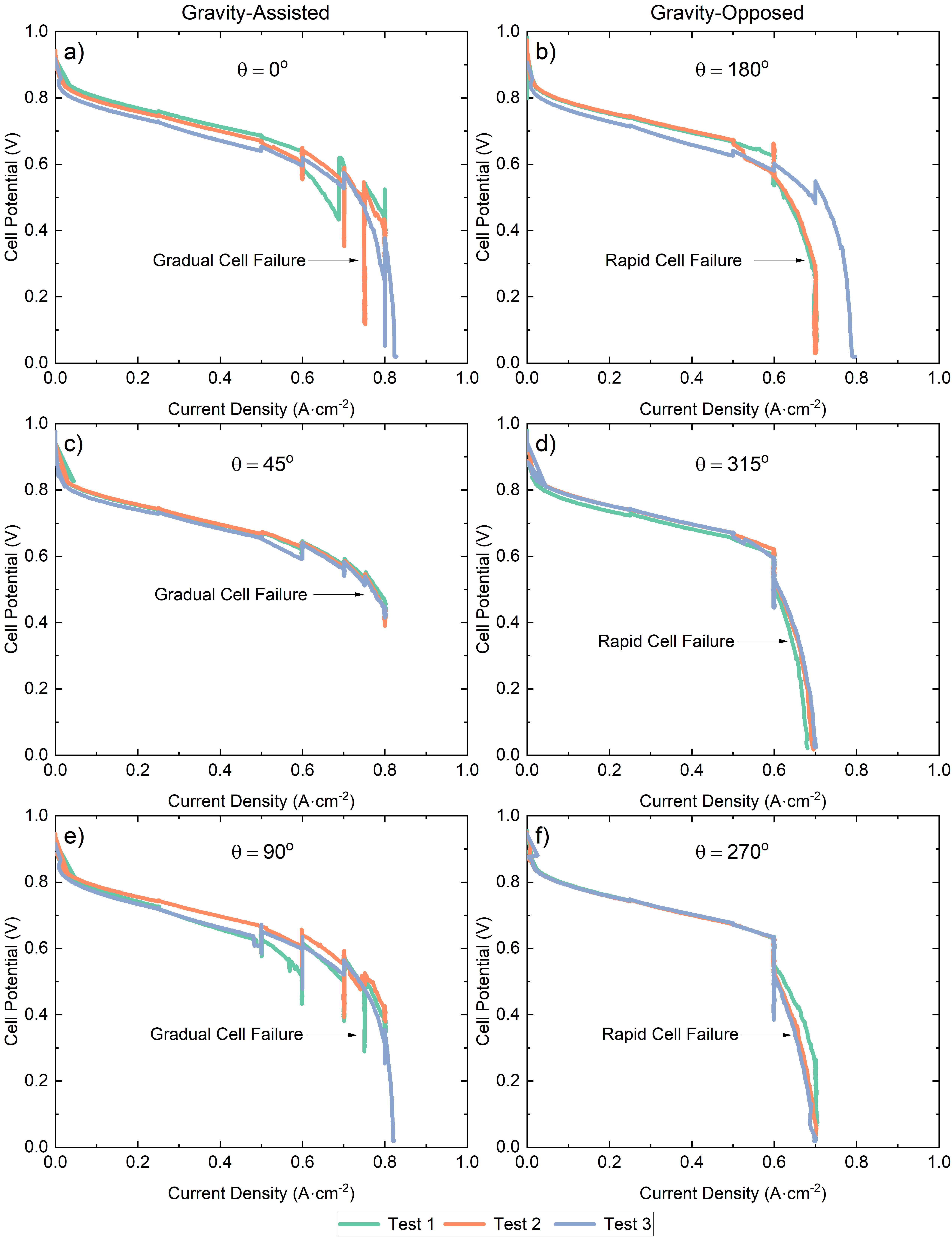


Figure S3: Sample transient polarization curves for a) 0º, b) 180º, c) 45º, d) 315º, e) 90º, f) 270º angles.

**Section S5. Supplementary Video 1: Liquid Water Transport During Fuell Cell Operation at 180°.**

The video has been published on an open-access repository:

Chadwick, E. A. (2025, March 13). Supplementary Video 1: Liquid Water Transport During Fuell Cell Operation at 180°. Zenodo. <https://doi.org/10.5281/zenodo.15016533>

References

1. Gostick, J. T. Versatile and efficient pore network extraction method using marker-based watershed segmentation. *Phys Rev E* **96**, 023307 (2017).
